# The novel leucine-rich repeat receptor-like kinase MRK1 regulates resistance to multiple stresses in tomato

**DOI:** 10.1093/hr/uhab088

**Published:** 2022-01-20

**Authors:** Qiaomei Ma, Zhangjian Hu, Zhuo Mao, Yuyang Mei, Shuxian Feng, Kai Shi

**Affiliations:** Department of Horticulture, Zhejiang University, 866 Yuhangtang Road, Hangzhou 310058, China; Department of Horticulture, Zhejiang University, 866 Yuhangtang Road, Hangzhou 310058, China; Department of Horticulture, Zhejiang University, 866 Yuhangtang Road, Hangzhou 310058, China; Department of Horticulture, Zhejiang University, 866 Yuhangtang Road, Hangzhou 310058, China; Department of Horticulture, Zhejiang University, 866 Yuhangtang Road, Hangzhou 310058, China; Department of Horticulture, Zhejiang University, 866 Yuhangtang Road, Hangzhou 310058, China

## Abstract

Leucine-rich repeat receptor-like kinases (LRR-RLKs) are ubiquitous in higher plants and act as receptors of extracellular signals to trigger multiple physiological processes. However, the functions of the majority of LRR-RLKs remain largely unknown, especially in tomato (*Solanum lycopersicum* L.). Here, we found that *MRK1* (*Multiple resistance-associated kinase 1*), encoding a novel tomato LRR-RLK, was significantly induced by temperature stresses and bacterial pathogen attacks. Knocking out *MRK1* impaired tolerance to both cold and heat stress, accompanied by decreased transcript levels of the master regulators *C-repeat binding factor 1* (*CBF1*) and *Heat shock transcription factor a-1a* (*HsfA1a*), respectively. In addition, *mrk1* mutants were hypersensitive to *Pseudomonas syringae* pv. *tomato* DC3000 and *Ralstonia solanacearum* and showed compromised pattern-triggered immunity (PTI) responses, as evidenced by decreased production of reactive oxygen species and reduced upregulation of PTI marker genes. Moreover, bimolecular fluorescence complementation, split-luciferase assays, and co-immunoprecipitation supported the formation of a complex of MRK1, FLS2, and Somatic embryogenesis receptor kinase (SERK3A/SERK3B) in a ligand-independent manner. This work demonstrates that tomato MRK1 is a novel positive regulator of multiple stress responses and may be a potential breeding target for improving crop stress resistance.

## Introduction

Climate change increases the frequency of various stresses such as extreme temperatures and microbial pathogen attacks. As sessile organisms, plants have to manage all such biotic and abiotic stresses at the same time. Over the course of evolution, plants have acquired multiple strategies to deal with adverse environmental conditions. These usually consist of three steps: stress signal perception, signal transduction, and activation of related gene expression, which contribute to protective or adaptive physiological responses [1]. Accurate signal perception is the first step by which plants stimulate appropriate defense responses. Plants employ a set of receptor-like kinases (RLKs) on the cell surface to detect diverse signals in the apoplast between the cells. The leucine-rich repeat RLKs (LRR-RLKs) are well studied and are one of the largest classes of RLKs. Nearly half of the estimated receptor kinases in *Arabidopsis* genomes possess LRR receptor domains, and there are 234 LRR-RLK genes in the tomato genome [2, 3]. LRR-RLKs contain three functional domains: an extracellular LRR region responsible for signal recognition, a transmembrane domain to immobilize the protein on the membrane, and a cytoplasmic protein kinase domain that participates in signal transduction by autophosphorylation and subsequent phosphorylation of specific substrates [4].

LRR-RLKs are ubiquitous in plants and play critical roles in responses to biotic and abiotic stresses [5, 6]. Several LRR-RLKs have been shown to participate in pathogen recognition, functioning as pattern recognition receptors (PRRs) to sense diverse pathogen- or damage-associated molecular patterns (PAMPs or DAMPs). Upon recognition, these PRRs trigger basal immunity and non-host resistance [7, 8]. In sensitive host plants, PRR-triggered immunity can effectively repulse the majority of virulent pathogens, contributing to basal immunity, which is also called pattern-triggered immunity (PTI) [7, 8]. PTI responses involve the production of reactive oxygen species (ROS), the activation of mitogen-activated protein kinase (MAPK), and the transcriptional reprogramming of immunity-associated genes [9–11]. The well-studied PRRs contain the bacterial flagellin receptors FLS2 (Flagellin sensing 2) and FLS3, the bacterial elongation factor Tu receptor EFR, the peptide Pep receptors PEPR1 and PEPR2, and the peptide phytosulfokine (PSK) receptor PSKR1 [12–15]. Interestingly, LRR-RLKs form complexes with each other to contribute to plant immunity. For example, the *Arabidopsis* LRR-RLK receptor BRASSINOSTEROID INSENSITIVE1-ASSOCIATED RECEPTOR KINASE (BAK1) functions as a co-receptor, forming complexes with FLS2 or EFR to promote ligand perception, which plays a role in resistance to *Pseudomonas syringae* and *Hyaloperonospora arabidopsidis* [16–18]. Similarly, RXEG1 (Receptor-like protein response to XEG1) forms a complex with BAK1 and SOBIR1 (Suppressor of BIR1–1) to transduce defense signals against *Phytophthora sojae* [19]. In addition, some LRR-RLKs have been found to participate in abiotic stress defense responses. Several studies have reported that LRR-RLKs can sense the change in membrane fluidity caused by abiotic stresses like cold and heat and activate the expression of stress-responsive genes [20–22]. For instance, GsLRPK from *Glycine soja* functions as a key regulator in cold tolerance [23]; OsCTB4a enhances rice cold tolerance [24]; and OsGIRL1 negatively regulates rice heat tolerance [25]. Despite these examples, the vast majority of LRR-RLKs have not been investigated, and their possible contributions to plant defense responses remain largely unknown, especially for those that function in both biotic and abiotic stresses.

Tomato (*Solanum lycopersicum* L.) is an economically important vegetable crop worldwide, and its production is threatened by temperature fluctuations and pathogen attacks, causing severe crop losses. Because tomato is a temperature-sensitive plant, temperatures lower than 12°C or higher than 35°C significantly impair its growth, resulting in up to 70% yield losses [26, 27]. Global climate change has markedly increased the frequency of temperature fluctuations, which adversely affect tomato productivity. Moreover, diseases caused by various bacterial pathogens frequently occur in tomato cultivation. In particular, *P. syringae* pv. *tomato* (*Pst* DC3000) and *Ralstonia solanacearum* are the two most important bacterial pathogens and cause bacterial leaf speck disease and root-borne bacterial wilt disease, respectively [28–30]. Characterization of the functions of LRR-RLKs in tomato is a prerequisite for breeding tomato cultivars with enhanced resistance to various stresses. In this study, through analyzing the expression patterns of 234 tomato LRR-RLK genes in response to temperature stress and bacterial pathogen inoculation, we identified a novel LRR-RLK from tomato, Solyc01g105080, and named it Multiple resistance-associated kinase 1 (*MRK1*). To gain deeper insight into the function of *MRK1* in response to multiple stresses, we generated two homozygous *mrk1* mutant lines using CRISPR/Cas9 and *MRK1* overexpression plants using transgenic overexpression approaches. Our results showed that *MRK1* plays a positive role in the resistance to both cold and heat stresses, as well as the PTI response, and it functions as a component in a complex with FLS2 and the co-receptor Somatic embryogenesis receptor kinase (SERK3A/SERK3B) to trigger immunity signaling. This study not only broadens our understanding of the role of LRR-RLKs in defense against multiple stresses but also highlights *MRK1* as an ideal gene for breeding tomato with resistance to multiple biotic and abiotic stresses.

## Results

### *MRK1* is induced by multiple stresses

**Figure 1:**
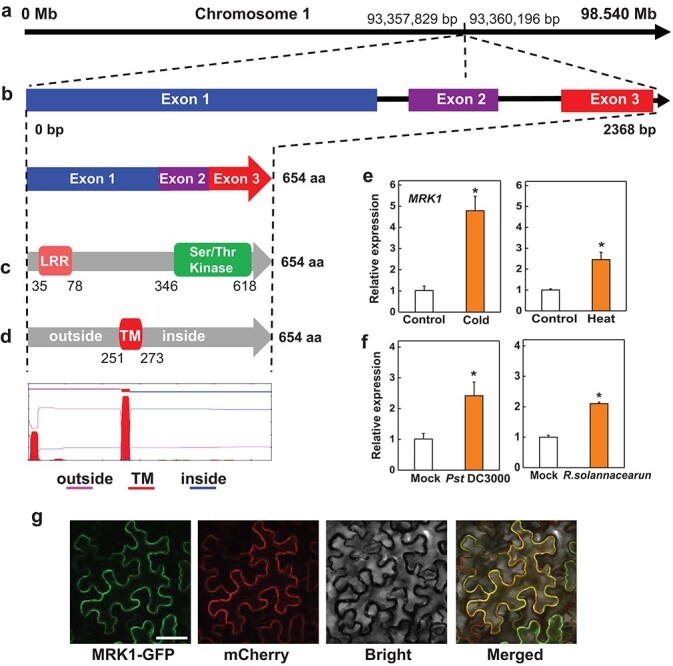
Bioinformatics and expression characteristics of tomato Multiple resistance-associated kinase 1 (*MRK1*). **a**, **b** The chromosomal locus and exon-intron structure of *MRK1*. Data were retrieved from the Solanaceae Genomics Network. **c** The functional domains of MRK1 determined using Pfam. **d** Illustration of the putative transmembrane region of MRK1 predicted by TMHMM V2.0. The expression profiles of *MRK1* in response to cold (4°C) and heat (45°C) stress (**e**) and *Pst* DC3000 and *R. solanacearum* inoculation (**f**) were quantified using qRT-PCR with *ACTIN2* as a normalization control. Samples were collected at 6 hours after exposure to cold or heat stress, at 12 hours after *Pst* DC3000 infection, and at 3 days after *R. solanacearum* inoculation. **h** Subcellular localization of MRK1. The tomato MRK1-GFP plasmid was transiently expressed in *N. benthamiana* leaves. The GFP and mCherry (a plasma membrane marker) signals were visualized by confocal microscopy at 48 h after infiltration. Bars = 50 μm. An asterisk indicates a significant difference between treatments (*P* < 0.05, Tukey’s test). The results in **e** and **f** are presented as mean values ± SD; *n* = 3. The experiment was performed three times with similar results.

The expression patterns of all tomato genes under temperature stress and bacterial pathogen attacks were inferred from tomato gene transcriptomic studies [31, 32]. We found that a gene (Solyc01g105080) annotated as a putative kinase was significantly upregulated in response to multiple stresses, and we subsequently named it Multiple resistance-associated kinase 1 (*MRK1*) (Table S1)*.* The MRK1 protein is predicted to be a transmembrane LRR-RLK by SMART and TMHMM V2.0 (Fig. 1a–d). We confirmed the expression of *MRK1* using quantitative real-time polymerase chain reaction (qRT-PCR). *MRK1* transcript levels increased 5- and 2.5-fold after exposure to cold and heat stress, respectively (Fig. 1e). Meanwhile, inoculation with the bacterial pathogens *Pst* DC3000 and *R. solanacearum* also significantly enhanced the expression of *MRK1* at 12 hours post-inoculation (hpi) and 3 days post-inoculation (dpi), respectively (Fig. 1f)*.* However, the expression of *MRK1* was not affected by inoculation with the necrotrophic fungal pathogen *Botrytis cinerea* (Fig. S1a).

We also analyzed the subcellular localization of tomato MRK1 by transiently expressing an MRK1-GFP fusion protein driven by the cauliflower mosaic virus 35S promoter in *Nicotiana benthamiana* leaves. As shown in Fig. 1g, the fluorescence signal was present mainly on the plasma membrane (Fig. 1g), suggesting that MRK1 is probably localized in the plasma membrane.

### Generation of *mrk1* mutants and *MRK1* overexpression plants in tomato

**Figure 2:**
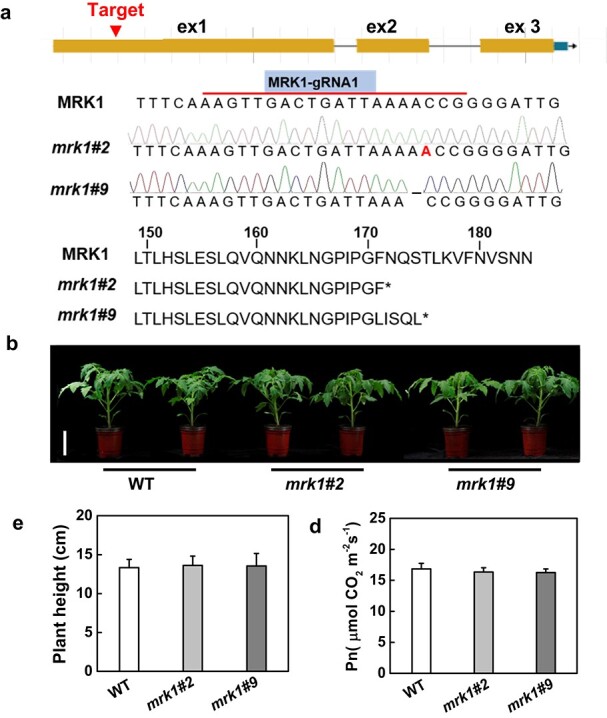
Generation of tomato *MRK1* mutants by CRISPR/Cas9. **a** Schematics show the guide-RNA (gRNA) target site in the first exon (ex) of *MRK1* and the missense mutations present in two *mrk1* mutant lines (*mrk1#2* and *mrk1#9*). The *mrk1#2* line has a 1-bp insert, and the *mrk1#9* line has a 1-bp deletion. The *mrk1* mutant lines have a premature stop codon at the 172^nd^ or 176^th^ amino acid of the MRK1 protein. **b** Plant phenotypes of WT plants and *mrk1* mutants at 4 weeks after germination. Bars = 8 cm. Plant height (**c**) and net photosynthetic rate, Pn, (**d**) were measured at the same time. An asterisk indicates a significant difference between treatments (*P* < 0.05, Tukey’s test). The results in **c** and **d** are presented as mean values ± SD; *n* = 8.

To study the potential role of *MRK1* in plant resistance, mutations in *MRK1* were generated by CRISPR/Cas9. The CRISPR-P web tool was used to design a guide RNA, *MRK1*-gRNA (AAGTTGACTGATTAAAACCG; Fig. 2a), that targeted the first exon of the *MRK1* gene. After transformation of the cultivar Condine Red, we obtained two homozygous mutant lines (*mrk1#2* and *mrk1#9*) (Fig. 2a). Line *mrk1#2* had a 1-bp insertion in *MRK1* that resulted in an early stop codon at the 172^nd^ amino acid of the protein, whereas line *mrk1#*9 had a 1-bp deletion in MRK1 that resulted in an early stop codon at the 176^th^ amino acid (Fig. 2a). The expression levels of *MRK1* and several other well-studied LRR-RLKs were also analyzed in *mrk1* mutants by qRT-PCR, and the results showed that knocking out *MRK1* only reduced the expression of *MRK1* (Fig. S2). In addition, we generated two *MRK1* overexpression lines, OE-*MRK1*#1 and OE-*MRK1*#3, with the 35S promoter using transgenic overexpression approaches (Fig. S3a). The growth patterns of the two *mrk1* mutant lines and the *MRK1* overexpression lines were indistinguishable from those of wild-type (WT) plants (Fig. 2b and Fig. S3b). Likewise, there were no obvious differences in plant height and net photosynthetic rate (Pn) between *mrk1* mutants and WT plants (Fig. 2c–d).

### MRK1 is critical for tomato tolerance to temperature stress

**Figure 3:**
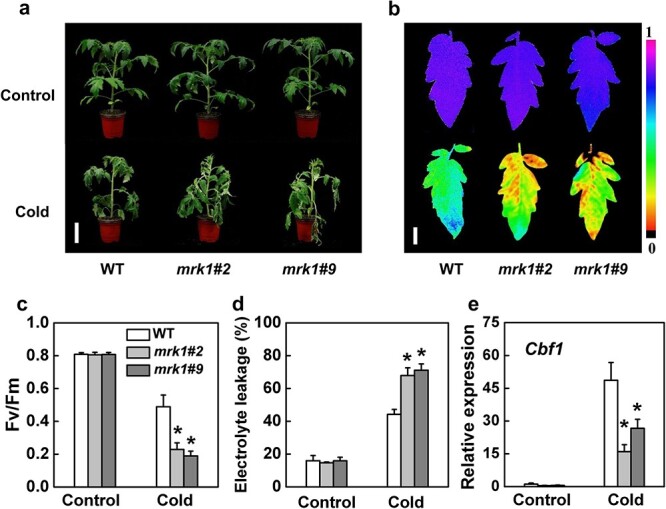
MRK1 positively regulates tomato cold tolerance. **a** Representative image of *mrk1* mutants and WT plants after exposure to control temperature (Control, 25°C) or cold temperature (Cold, 4°C) for 7 days. Bars = 8 cm. **b, c** The maximum photochemical efficiency of PSII (Fv/Fm) of WT plants and *mrk1* mutants after 7 days at different temperatures. The color gradient scale (**b)** at the right indicates the magnitude of the fluorescence signal represented by each color. Bars = 1 cm. **d** Relative electrolyte leakage of WT and *mrk1* leaves after 7 days at 25°C or 4°C. **e**  *CBF1* expression in tomato leaves was assessed by qRT-PCR after 6 h at 4°C. An asterisk indicates a significant difference between treatments (*P* < 0.05, Tukey’s test). The results in **c** to **e** are presented as mean values ± SD; *n* = 5. These experiments were performed twice with similar results.

To investigate the function of *MRK1* in cold tolerance, *mrk1* mutants and WT plants were exposed to 4°C. We found that the *mrk1* mutants exhibited increased sensitivity to cold stress, as shown by changes in plant phenotype, reduced maximum photochemical efficiency of PSII (Fv/Fm), and increased relative electrolyte leakage (REL, an indicator of membrane permeability) (Figs. 3a–d). Previous studies have shown that the transcription factor C-repeat binding factor (CBF) pathway plays a critical role in cold stress response [33]. In this study, transcripts of *CBF1* increased significantly in WT plants but were greatly suppressed in *mrk1* mutants upon exposure to cold stress (Fig. 3e). These results suggest that *MRK1* has a positive role in cold tolerance and is associated with the CBF pathway.

**Figure 4:**
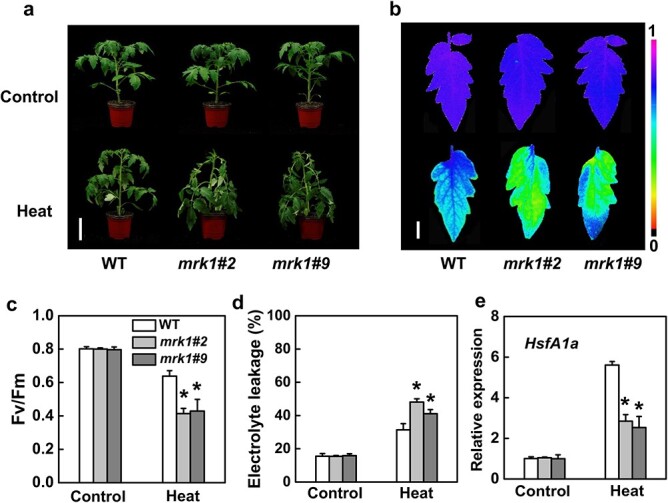
MRK1 positively regulates tomato thermotolerance. **a** Representative image of *mrk1* mutants and WT plants after exposure to control temperature (Control, 25°C) or high temperature (Heat, 45°C) for 12 h. Bars = 8 cm. **b, c** The maximum photochemical efficiency of PSII (Fv/Fm) of WT plants and *mrk1* mutants after 12 h at different temperatures. The color gradient scale (**b)** at the right indicates the magnitude of the fluorescence signal represented by each color. Bars = 1 cm. **d** Relative electrolyte leakage of WT and *mrk1* leaves after 12 h at 25°C or 45°C. **e**  *HsfA1a* expression in tomato leaves was assessed by qRT-PCR after 12 h at 45°C. An asterisk indicates a significant difference between treatments (*P* < 0.05, Tukey’s test). The results in **c** and **e** are presented as mean values ± SD; *n* = 5. These experiments were performed twice with similar results.

**Figure 5:**
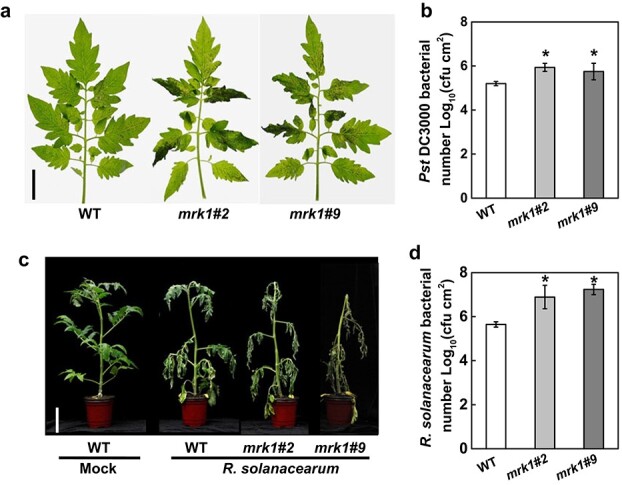
MRK1 positively regulates tomato disease resistance to *P. syringae* pv. *tomato* (*Pst* DC3000) and *R. solanacearum* pathogens. **a** Disease symptoms of *mrk1* mutants and WT plants photographed at 5 days post inoculation (dpi) with *Pst* DC3000. Bars = 2 cm. **b**  *Pst* DC3000 bacterial populations were assessed at 3 dpi. **c** The phenotypes of tomato plants at 10 dpi with *R. solanacearum* and *R. solanacearum* bacterial populations (**d)** in plants assessed at 10 dpi. Bars = 8 cm. An asterisk indicates a significant difference between treatments (*P* < 0.05, Tukey’s test). The results in **b** and **d** are presented as mean values ± SD; *n* = 5. These experiments were performed three times with similar results.

Next, *mrk1* mutants and WT plants were exposed to 45°C to investigate the role of *MRK1* in thermotolerance. Crucially, *mrk1* mutants also showed a higher susceptibility to heat stress. Compared with WT plants, *mrk1* mutants exhibited a notable decrease in Fv/Fm and a significant increase in REL (Fig. 4a–c). Heat shock transcription factor a-1a (HsfA1a) has been identified as a master regulator of the heat-shock response in tomato plants [34]. The induction of *HsfA1a* expression by heat stress was largely suppressed in *mrk1* mutants (Fig. 4e). These results demonstrate that *MRK1* is critical for plant tolerance to extreme temperature stress.

The role of *MRK1* in response to extreme temperature stress was further evaluated by analyzing the tolerance of OE-*MRK1* plants. However, the overexpression of *MRK1* did not further enhance tolerance to either cold or heat stress (Fig. S3c–d).

### MRK1 is required for resistance to bacterial disease

The expression of *MRK1* was strongly induced by inoculation with the bacterial pathogens *Pst* DC3000 and *R. solanacearum* (Fig. 1f). Both *mrk1* mutant lines exhibited increased disease symptoms compared with WT plants at 5 dpi with *Pst* DC3000, as reflected by increased chlorosis and necrosis of tomato leaves (Fig. 5a). This phenotype was consistent with greatly increased bacterial growth in *mrk1* mutant leaves compared with WT leaves (Fig. 5b). Similarly, *mrk1* mutants were more susceptible to *R. solanacearum*. The *mrk1* mutants showed more severe disease symptoms than WT plants at 10 dpi with *R. solanacearum* (Fig. 5c), with significantly higher bacterial growth on leaves (Fig. 5d). The role of *MRK1* in anti-bacterial immunity was further evaluated by analyzing the susceptibility of OE-*MRK1* plants to *Pst* DC3000 and *R. solanacearum*. However, overexpression of *MRK1* was unable to improve the resistance to these bacterial diseases compared with that of wild-type controls (Fig. S3e–h).

To investigate the function of *MRK1* in plant immunity to pathogens other than bacteria, *mrk1* mutants were used to evaluate the role of *MRK1* in resistance to the necrotrophic fungal pathogen *B. cinerea.* The *mrk1* mutants were as sensitive as WT plants to *B. cinerea*, suggesting that *MRK1* has no evident function in defense against this necrotrophic fungus (Fig. S1b–c). Together, these results indicate that *MRK1* plays a positive role in tomato anti-bacterial immunity.

### MRK1 modulates PTI responses

**Figure 6:**
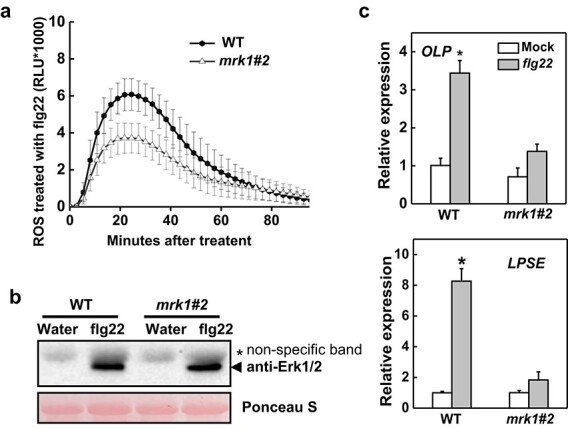
Effects of *MRK1* knockout on the pathogen-associated molecular pattern (PAMP)-induced immunity (PTI) response. **a** ROS production in *mrk1* mutants. Leaf discs from *mrk1* mutants and WT plants were treated with 100 nM flg22, and the production of ROS is expressed as relative light units (RLU) over a period of 80 minutes after elicitation. Values are means ± SD of three independent experiments, each with six leaf discs. One-way analysis of variance (ANOVA) followed by Tukey’s honestly significant difference post hoc test (*p* < 0.05) was performed at 23 min (peak readout) after elicitation. There was an obvious decrease in ROS production of *mrk1* mutants compared with WT plants. **b** MAPK activation in *mrk1* mutants. Leaf discs from *mrk1* mutants and WT plants were treated with water or 100 nM flg22 for 10 min. Proteins were extracted and subjected to immunoblotting using an anti-pMAPK antibody, which detects the phosphorylated MAPKs. Ponceau S staining was used to estimate equal loading in each lane. Similar results were observed in a second independent experiment. **c** Upregulation of the PTI-responsive genes *Osmotin-like protein* (*OLP*) and *Lipid particle serine esterase* (*LPSE*). Relative expression of *OLP* and *LPSE* was evaluated at 1 h post treatment with 100 nM flg22 in *mrk1* mutants and WT plants. Relative expression levels were compared with those of the WT mock control (set to 1) using qRT-PCR analysis with *ACTIN2* as a normalization control. Values are means ± SD of three independent experiments, each with three samples from six plants. An asterisk indicates a significant difference between treatments (*P* < 0.05, Tukey’s test). These experiments were performed twice with similar results.

PTI response is an important component of plant basal immunity, which can repel most virulent pathogens. To assess whether MRK1 is related to PTI responses, we monitored the expression of *MRK1* after treatment with 100 nM flg22. The results showed that *MRK1* transcripts increased at 1 h after treatment (Fig. S4). Notably, ROS production and activation of MAPK cascades are two typical early PTI-related responses in plants [7, 10]. To assess whether MRK1 is involved in early PTI events, we performed ROS assays with flg22 in *mrk1* and WT leaves. Because both of the *mrk1* mutant lines showed similar susceptibility to *Pst* DC3000 and *R. solanacearum*, most subsequent experiments focused on the *mrk1#2* line. PAMP-triggered ROS production was strongly reduced in *mrk1* mutants compared with WT plants (Fig. 6a). However, *mrk1* mutants displayed the same level of MAPK activation as the WT after treatment with flg22 (Fig. 6b). Together, these results illustrate that MRK1 is required for the ROS burst but not for MAPK activation.

Genes encoding Osmotin-like protein (OLP) and Lipid particle serine esterase (LPSE) are well known as PTI-specific markers in tomato [35]. To further examine the function of MRK1 in late PTI responses, we observed the expression of *OLP* and *LPSE* after treatment with flg22 (Fig. 6c). At 1 h after PAMP treatment, *mrk1* mutants showed less change in *OLP* and *LPSE* expression levels than WT plants. Together, these results indicate that MRK1 positively regulates several PTI responses.

### MRK1 associates with FLS2 and SERK3A/SERK3B in a ligand-independent manner

**Figure 7:**
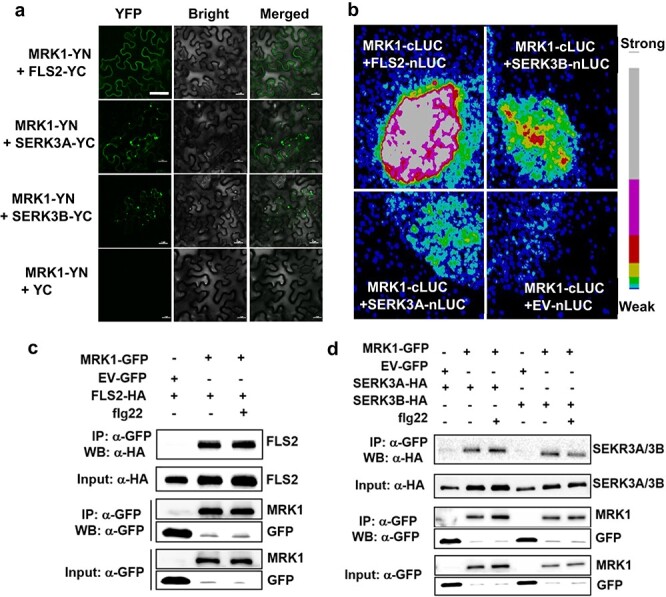
MRK1 associates with FLS2 and SERK3A/SERK3B in a complex. **a** Bimolecular fluorescence complementation (BiFC) assays showing MRK1 interaction with FLS2 and SERK3A/SERK3B. MRK1-YFP^N^ and FLS2-YFP^C^ or SERK3A/SERK3B-YFP^C^ were co-transfected into *N. benthamiana* leaves. The YFP fluorescence was visualized under a confocal microscope at 2 d after transfection. At least two independent experiments were performed with similar results. Bars = 50 μm. **b** Split-luciferase (LUC) assays showing the interactions of MRK1 with FLS2 and SERK3A/SERK3B. MRK1-nLUC and FLS2-cLUC or SERK3A/SERK3B-cLUC were co-transfected into *N. benthamiana* leaves. The signal was visualized using a Photek camera. The pseudocolor bar indicates the range of luminescence intensity. These experiments were repeated three times with similar results. **c, d** Co-immunoprecipitation of MRK1 with FLS2 or SERK3A/SERK3B proteins in *N. benthamiana.* Proteins were extracted from *N. benthamiana* leaves expressing MRK1-GFP in combination with FLS2-HA (**c**) or SERK3A/SERK3B-HA (**d**) and subjected to immunoprecipitation (IP) with GFP-trap beads followed by immunoblotting with anti-HA antibody. EV-GFP was used as a negative control. These experiments were performed twice with similar results.

The above results indicate that MRK1 acts upstream of the ROS burst in the PTI responses triggered by flg22. Thus, we considered the possibility that MRK1 associates with FLS2 as a complex, similar to previous reports on FLS2 and WAK1 in tomato [36]. First, bimolecular fluorescence complementation (BiFC) assays were used to verify the molecular interactions of MRK1 with FLS2 or the co-receptor SERK3A/SERK3B (an ortholog of *Arabidopsis* BAK1). YFP fluorescence signals showed that MRK1 interacted with FLS2 at the plasma membrane and interacted with SERK3A/SERK3B in the cytoplasm (Fig. 7a). Next, the interactions were evaluated by a split-luciferase assay, and the luciferase signal showed that MRK1 associated with both FLS2 and SERK3A/SERK3B (Fig. 7b).

Next, co-immunoprecipitation (CoIP) assays were used to determine whether the molecular interaction of MRK1 with FLS2 or SERK3A/SERK3B was affected by the presence of flg22. MRK1-GFP was co-expressed with HA epitope-tagged FLS2 or SERK3A/SERK3B in *N. benthamiana*. Equal amounts of samples were used for immunoprecipitation with GFP-Trap beads, and FLS2-HA or SERK3A/SERK3B-HA was detected by anti-HA immunoblotting. FLS2 was detected in both mock- and flg22-treated samples (Fig. 7c). Likewise, SERK3A/SERK3B was also detected in the MRK1 immunoprecipitate with and without flg22 (Fig. 7d). These observations demonstrate that MRK1 forms a complex with FLS2 and SERK3A/SERK3B in a ligand-independent manner.

## Discussion

In the natural environment, plants are frequently exposed to a range of environmental challenges involving biotic and abiotic stress, and they must therefore cope with multiple stresses at the same time. In plants, LRR-RLKs comprise a large gene family and modulate multiple plant processes, including responses to biotic and abiotic stress [5]. Genetic analyses have identified the roles of a small fraction of LRR-RLKs, but most have yet to be functionally defined, especially those that are involved in multiple signaling pathways [5, 37]. Here, we identified a novel LRR-RLK gene *MRK1* from tomato that was upregulated by temperature stress and bacterial pathogen inoculation. Our results demonstrate that MRK1 positively regulates tolerance to cold and heat by regulating the transcripts of master transcription factors in temperature stress. MRK1 also associates in a complex with FLS2 and SERK3A/SERK3B to modulate the flagellin-induced PTI response.

In this study, a reverse genetics approach identified two independent *mrk1* mutants with hypersensitivity to cold and heat stress and to the bacterial pathogens *Pst* DC3000 and *R. solanacearum* (Figs. 3–5) but with a sensitivity to the fungal pathogen *B. cinerea* similar to that of the WT (Fig. S1). However, overexpression of *MRK1* with the 35S promoter did not further enhance resistance to these stresses. One possibility is that the expression of *MRK1* in plants has been upregulated by various stresses, and its overexpression cannot further increase plant resistance. It is also possible that transgenic overexpression with the 35S promoter is an artificial process that cannot completely simulate the native physiological process in plants; the gene needs to be studied further with its native promoter. These results indicate that MRK1 is critical for tolerance to cold and heat and for resistance to hemi-biotrophic bacteria but not for tolerance to the necrotrophic fungus *B. cinerea*. Similarly, the *Arabidopsis* LRR-RLK gene *PHLOEM INTERCALATED WITH XYLEM-like 1* (*AtPXL1*) has been shown to play a positive role in the regulation of tolerance to both cold and heat stress [38], but its function in immunity remains unclear. Several LRR-RLKs have been shown to participate in responses to stress in tomato. For instance, FLS2 recognizes the flg22 peptide derived from *P. syringae* to trigger the immunity response [13], and EFR and PSKR1 positively regulate tomato immunity against *R. solanacearum* and *B. cinerea*, respectively [12, 39]. Based on the studies above, it appears that these LRR-RLKs only regulate the response to a single stress in tomato, whereas MRK1 identified here regulates the responses to multiple stresses. Furthermore, we found that mutation of *MRK1* did not impair the growth of tomato plants (Fig. 2). In conclusion, tomato LRR-RLK MRK1 appears to be a potentially ideal candidate for genetic engineering programs aimed at generating multiply resistant materials.

Plants have evolved a complex adaptation process in response to temperature stress [40]. Previous studies have shown that CBFs are master transcription factors in response to cold stress, activating the transcription of cold-regulated (COR) genes like *WRKY6* and *PYL6* [41]. HsfA1a can regulate most heat shock genes by binding to the heat shock element (HSE) motif under heat stress [42]. The LRR-RLK gene *GsLRPK* enhances plant cold tolerance by triggering the expression of CBFs [23]. Therefore, MRK1 possibly regulates plant cold and heat tolerance by modulating the expression of cold- and heat-inducible marker genes. Consistent with this assumption, we found that knocking out *MRK1* suppressed the upregulation of *CBF1* and *HsfA1a*, leading to compromised cold and heat tolerance, respectively (Figs. 3–4). However, the mechanisms by which transmembrane MRK1 regulates the expression of these two nuclear-localized master transcription factors remain unknown. It is possible that MRK1 may regulate the expression of these two genes through phosphorylation or transcription events, which need to be addressed in the future.

In this study, we found that the increased sensitivity of *mrk1* mutants to bacterial disease was related to a defective PTI response (Fig. 6). The flg22-induced production of ROS was greatly reduced in *mrk1* mutants. Moreover, upregulation of the PTI-responsive genes *OLP* and *LPSE* was greatly suppressed in *mrk1* mutants compared with WT plants. By contrast, the activation of MAPKs upon flg22 elicitation occurred at the same level in *mrk1* mutants and WT plants. These observations demonstrate that MRK1 is essential for the complete activation of both early and late PTI responses. Likewise, the LRR-RLK gene *IMPAIRED OOMYCETE SUSCEPTIBILITY1* (*IOS1*) is essential for activation of some early and late PTI responses in *Arabidopsis* [43]. However, *ios1* mutants exhibited reduced MAPK activation and reduced upregulation of *FLG22-INDUCED RECEPTOR-LIKE KINASE1* (*FRK1*), one of the PTI marker genes, but showed no defects in ROS production upon PAMP treatment, indicating that these two positive regulators of PTI have different mechanisms.

MRK1 is an LRR-RLK gene whose importance in flg22-triggered PTI upstream of the ROS burst and PTI marker gene expression was genetically clarified in the current study. These findings raised the possibility that MRK1 might form a part of PRR complexes for the recognition of bacterial PAMPs. We therefore tested this hypothesis and found that MRK1 does interact with FLS2 and the co-receptor SERK3A/SERK3B, but in an flg22-independent manner (Fig. 7). This is reminiscent of IOS1, an LRR-RLK from *Arabidopsis* that was found to associate with FLS2 and BAK1 to regulate the PTI response; elicitation by flg22 also does not significantly affect this association [43]. It is possible that MRK1, like IOS1, may play a positive role in FLS2-BAK1 complex formation. Based on our observations, we propose that MRK1 acts as a component of the FLS2 and SERK3A/SERK3B complex to modulate PTI responses.

In addition, salicylic acid (SA)-mediated signaling is one of the most important components of plant immunity for repelling microbial pathogens [44]. However, in the present study of plant defense against *Pst* DC3000 in tomato, knockout of *MRK1* did not impair SA accumulation or expression of the SA-responsive defense genes *PR1* and *PR4*, suggesting that *MRK1*-mediated defense may be independent of SA (Fig. S5). A previous study also showed that silencing *CaLRR-RLK1* in pepper did not affect the expression of the SA signaling-associated genes *CaPR1* and *CaNPR1* during *R. solanacearum* infection [45]. By contrast, negative regulation of cell death (an important response in plant immunity) by BAK1 and BAK1-LIKE1 (BKK1) was dependent on the SA pathway in *Arabidopsis* [46], and PSKR1 negatively regulated plant resistance to *Pst DC3000* via the SA-mediated signaling pathway [47]. The above results suggest that the mechanisms by which LRR-RLKs regulate immunity differ among different plant pathosystems.

In conclusion, we identified the novel LRR-RLK *MRK1*, which was induced by several abiotic and biotic stresses. We demonstrated that *MRK1* positively regulated cold and heat tolerance, perhaps by modulating the expression of the master regulator genes *CBF1* and *HsfA1a*, respectively. In addition, *MRK1* also plays a positive role in anti-bacterial immunity and acts as a component of an FLS2 and SERK3A/SERK3B complex. This research provides insights into the complex structure of LRR-RLK-mediated signaling systems and provides a new target for the breeding of tomato with enhanced resistance to multiple stresses.

## Materials and methods

### Plant materials and growth conditions

To knock out the *MRK1* gene in tomato, a guide RNA (AAGTTGACTGATTAAAACCG) targeting the first exon of *MRK1* was designed using CRISPR-P (http://crispr.hzau.edu.cn/CRISPR2/). Next, the gRNA was cloned into a Cas 9-expression binary vector (pCAMBIA1301) as described previously [31]. *Agrobacterium* cells containing the target construct were used for transformation of the tomato cultivar Condine Red (CR). Genomic DNA was extracted from each transgenic plant by the CTAB method, and the genomic regions spanning the guide RNA were amplified and then sequenced at Zhejiang Sunya Biotech Co., Ltd. *MRK1* overexpression tomato lines were generated by PCR-amplifying the coding sequence of *MRK1* and inserting it into the pGWB417 vector under the control of the CaMV 35S promoter with an MYC epitope tag at the C terminus. The confirmed plasmids were transformed into tomato CR by *Agrobacterium tumefaciens*-mediated cotyledon tissue culture. After confirmation by western blotting, two independent overexpression lines were selected for experiments.

Tomato plants were grown in a plant growth room under a 12-h light/dark photoperiod, 500 μmol m^−2^ s^−1^ photosynthetic photon flux density, 25/21°C (day/night) temperatures, and 85% relative humidity. Tomato plants were used for experiments at the five-leaf stage. *N. benthamiana* plants were grown under similar conditions. The net photosynthetic rate (Pn) was assessed with an LI-6400 photosynthesis system (LI-COR, Lincoln, NE, USA).

### Subcellular localization

*Agrobacterium* GV3101 strains carrying the 35S:MRK1-GFP vector and the FLS2-mCherry vector (as a plasma membrane marker) were used to perform transient expression in *N. benthamiana* as previously described [12]. At 48 h after infiltration, the fluorescence signals were detected with a Zeiss LSM 780 confocal microscope (Zeiss, Germany) according to a previously described method [48].

### Cold and heat tolerance assays

For the cold and heat stress treatments, tomato plants were exposed to temperatures of 4°C and 45°C, respectively, under 500 μmol m^−2^ s^−1^ photosynthetic photon flux density with a 12/12 h (light/dark) cycle and 85% relative humidity in controlled-environment growth chambers (Zhejiang Qiushi Artificial Environment, China).

Relative electrolyte leakage (REL), which indicates membrane permeability, was evaluated as described previously [49]. The maximum quantum yield of PSII (Fv/Fm) was measured with an Imaging-PAM (IMAG-MAXI; Heinz Walz).

### Pathogen inoculation and sensitivity assays

*P. syringae* pv. *tomato* DC3000 (*Pst* DC3000) was cultured at 28°C in King’s B solid medium with 25 mg mL^−1^ rifampicin. The bacterial infection with *Pst* DC3000 was carried out according to previously described methods [50]. Disease severity was evaluated by assessing the bacterial population at 3 d post-inoculation (dpi) and evaluating the plant phenotypes at 5 dpi. *R. solanacearum* was cultured in Casamino Peptone Agar (CPG) at 28°C. The inoculation with *R. solanacearum* was performed as described previously [51]_._  *In planta* stem colonization with *R. solanacearum* was measured at 10 dpi as described previously [51].

### RNA extraction and transcript analysis

Total RNA was extracted from leaves using RNA extraction kits (Easy-do Biotech Co., Ltd., China) and reverse transcribed using a HiScript II Q RT SuperMix for qPCR kit (Vazyme Biotech Co., Ltd., China) following the manufacturer’s instructions. The AceQ qPCR SYBR Green Master Mix Kit (Vazyme Biotech Co., Ltd., China) was used to perform real time-quantitative PCR (RT-qPCR) assays on a LightCycler 480 II detection system (Roche, Germany) as described previously [52]. The housekeeping gene *ACTIN2* was used as the internal reference gene. Sequences of primer pairs are listed in Table S1.

### Reactive oxygen species (ROS) assay

ROS production was measured according to a previously described method [36]. After treatment with water or flg22, ROS production was then measured with a Synergy 2 microplate reader (BioTek).

### Mitogen-activated protein kinase (MAPK) phosphorylation assay

The activation of MAPKs was measured according to a previously described method [36]. Leaf discs were used to perform the experiment after allowing the wound response to subside, and samples were collected after treatment with 10 nM flg22. Next, the total protein was extracted, and MAPK phosphorylation was assessed with an anti-phospho-p44/42 MAPK (Erk1/2) antibody (anti-pMAPK; Cell Signaling).

### Bimolecular fluorescence complementation assay

Bimolecular fluorescence complementation (BiFC) assays were carried out as described previously [12]. In brief, *MRK1*, *FLS2*, and *SERK3A/SERK3B* genes were cloned into the BiFC vectors p2YN and p2YC, which were generously provided by C. Mao (Zhejiang University, China). At 48 h after infiltration of *N. benthamiana* leaves with *Agrobacterium* strains, samples were examined using a Zeiss LSM 780 confocal microscope (520 to 560 nm wavelengths).

### Split-luciferase assay

pCAMBIA-GW-nLUC and pCAMBIA-GW-cLUC were used for the split-luciferase assay and were provided by Y. Liang (Zhejiang University, China). At 48 h after infiltration with *Agrobacterium* strains, *N. benthamiana* leaves were incubated with 1 mM luciferin (MedChemExpress, USA) for 10 min. A Photek camera (HRPCS5, Photek) was then used to capture signals and images.

### Co-immunoprecipitation

Co-immunoprecipitation (CoIP) was performed as described previously [36]. *Agrobacterium* strains carrying the given vectors with *MRK1*, *FLS2* and *SERK3A/SERK3B* or *GFP* were infiltrated into *N. benthamiana* leaves. About 48 h later, leaves were treated with 100 nM flg22 or buffer for 10 minutes before harvest, and the total protein was then extracted from the *N. benthamiana* tissue using extraction buffer.

Soluble proteins of each sample were incubated with GFP-Trap beads (Chromotek) for 2 h at 4°C, then washed three times with extraction buffer. In order to analyze the immunoprecipitated proteins, immunoblotting was performed with anti-GFP or anti-HA antibodies.

## Supplementary Material

Web_Material_uhab088

## Data Availability

Data sharing is not applicable to this article because no data sets were generated or analyzed in this study.

## Statistical analysis

At least three independent biological replicates from different plants were performed for each experiment. Unless otherwise stated, experiments were performed three times independently. The data obtained were subjected to analysis of variance using SAS software version 8 (SAS Institute) and tested for significance using one-way analysis of variance (ANOVA) followed by Tukey’s honestly significant difference post hoc test.

## Accession numbers

Sequence data from this article can be found at the Sol genomics network database (http://solgenomics.net/) under the following accession numbers: *MRK1* (Solyc01g105080), *FLS2* (Solyc02g070890), *SERK3A* (Solyc10g047140), *SERK3B* (Solyc01g104970), *CBF1* (Solyc03g026280), *HsfA1a* (Solyc08g005170), *OLP* (Solyc11g044390), *LPSE* (Solyc04g077180), *PAL4* (Solyc09g007890), *PR1* (Solyc01g106620), *PR4* (Solyc00g174340) and *ACTIN2* (Solyc03g078400).

## References

[1] Ahanger MA, Akram N, Ashraf M et al. Signal transduction and biotechnology in response to environmental stresses. Biol Plant. 2017;61:401–16.

[2] Wei Z, Wang J, Yang S et al. Identification and expression analysis of the *LRR-RLK* gene family in tomato (*Solanum lycopersicum*) Heinz 1706. Genome. 2015;58:121–34. 26207619 10.1139/gen-2015-0035

[3] Shiu SH, Bleecker AB. Receptor-like kinases from Arabidopsis form a monophyletic gene family related to animal receptor kinases. PNAS. 2001;98:10763–8. 11526204 10.1073/pnas.181141598 PMC58549

[4] He X, Feng T, Zhang D et al. Identification and comprehensive analysis of the characteristics and roles of leucine-rich repeat receptor-like protein kinase (LRR-RLK) genes in *sedum alfredii* Hance responding to cadmium stress. Ecotoxicol Environ Saf. 2019;167:95–106. 30312890 10.1016/j.ecoenv.2018.09.122

[5] Ye Y, Ding Y, Jiang Q et al. The role of receptor-like protein kinases (*RLKs*) in abiotic stress response in plants. Plant Cell Rep. 2017;36:235–42. 27933379 10.1007/s00299-016-2084-x

[6] Oh E, Lee Y, Chae WB et al. Biochemical analysis of the role of leucine-rich repeat receptor-like kinases and the carboxy-terminus of receptor kinases in regulating kinase activity in *Arabidopsis thaliana* and *Brassica oleracea*. Molecules. 2018;23:236. 10.3390/molecules23010236 PMC6017770 29361797

[7] Couto D, Zipfel C. Regulation of pattern recognition receptor signalling in plants. Nat Rev Immunol. 2016;16:537–52. 27477127 10.1038/nri.2016.77

[8] Yu X, Feng B, He P et al. From chaos to harmony: responses and signaling upon microbial pattern recognition. Annu Rev Phytopathol. 2017;55:109–37. 28525309 10.1146/annurev-phyto-080516-035649 PMC6240913

[9] Jia Y, Martin GB. Rapid transcript accumulation of pathogenesis-related genes during an incompatible interaction in bacterial speck disease-resistant tomato plants. Plant Mol Biol. 1999;40:455–65. 10437829 10.1023/a:1006213324555

[10] Zipfel C . Plant pattern-recognition receptors. Trends Immunol. 2014;35:345–51. 24946686 10.1016/j.it.2014.05.004

[11] Li B, Meng X, Shan L et al. Transcriptional regulation of pattern-triggered immunity in plants. Cell Host Microbe. 2016;19:641–50. 27173932 10.1016/j.chom.2016.04.011 PMC5049704

[12] Zhang H, Hu Z, Lei C et al. A plant phytosulfokine peptide initiates auxin-dependent immunity through cytosolic Ca^2+^ signaling in tomato. Plant Cell. 2018;30:652–67. 29511053 10.1105/tpc.17.00537 PMC5894845

[13] Roberts R, Liu AE, Wan L et al. Molecular characterization of differences between the tomato immune receptors flagellin sensing 3 and flagellin sensing 2. Plant Physiol. 2020;183:1825–37. 32503903 10.1104/pp.20.00184 PMC7401135

[14] Mitre LK, Teixeira-Silva NS, Rybak K et al. The Arabidopsis immune receptor EFR increases resistance to the bacterial pathogens *Xanthomonas* and *Xylella* in transgenic sweet orange. Plant Biotechnol J. 2021;19:1294–6. 33991397 10.1111/pbi.13629 PMC8313127

[15] Yamaguchi Y, Huffaker A, Bryan AC et al. PEPR2 is a second receptor for the pep1 and pep2 peptides and contributes to defense responses in Arabidopsis. Plant Cell. 2010;22:508–22. 20179141 10.1105/tpc.109.068874 PMC2845411

[16] Chinchilla D, Zipfel C, Robatzek S et al. A flagellin-induced complex of the receptor FLS2 and BAK1 initiates plant defence. Nature. 2007;448:497–500. 17625569 10.1038/nature05999

[17] Heese A, Hann DR, Gimenze-Ibanz S et al. The receptor-like kinase SERK3/BAK1 is a central regulator of innate immunity in plants. PNAS. 2007;104:12217–22. 17626179 10.1073/pnas.0705306104 PMC1924592

[18] Roux M, Schwessinger B, Albrecht C et al. The Arabidopsis leucine-rich repeat receptor-like kinases BAK1/SERK3 and BKK1/SERK4 are required for innate immunity to hemibiotrophic and biotrophic pathogens. Plant Cell. 2011;23:2440–55. 21693696 10.1105/tpc.111.084301 PMC3160018

[19] Wang Y, Xu Y, Sun Y et al. Leucine-rich repeat receptor-like gene screen reveals that *Nicotiana* RXEG1 regulates glycoside hydrolase 12 MAMP detection. Nat Commun. 2018;9:594. 29426870 10.1038/s41467-018-03010-8 PMC5807360

[20] Falcone DL, Ogas JP, Somerville CR. Regulation of membrane fatty acid composition by temperature in mutants of Arabidopsis with alterations in membrane lipid composition. BMC Plant Biol. 2004;4:17. 15377388 10.1186/1471-2229-4-17 PMC524174

[21] Zhu J . Abiotic stress signaling and responses in plants. Cell. 2016;167:313–24. 27716505 10.1016/j.cell.2016.08.029 PMC5104190

[22] Sangwan V, Orvar BL, Beyerly J et al. Opposite changes in membrane fluidity mimic cold and heat stress activation of distinct plant MAP kinase pathways. Plant J. 2002;31:629–38. 12207652 10.1046/j.1365-313x.2002.01384.x

[23] Yang L, Wu K, Gao P et al. GsLRPK, a novel cold-activated leucine-rich repeat receptor-like protein kinase from *Glycine soja*, is a positive regulator to cold stress tolerance. Plant Sci. 2014;215–216:19–28. 10.1016/j.plantsci.2013.10.009 24388511

[24] Zhang Z, Li J, Pan Y et al. Natural variation in *CTB4a* enhances rice adaptation to cold habitats. Nat Commun. 2017;8:14788. 28332574 10.1038/ncomms14788 PMC5376651

[25] Park S, Moon JC, Park YC et al. Molecular dissection of the response of a rice leucine-rich repeat receptor-like kinase (LRR-RLK) gene to abiotic stresses. J Plant Physiol. 2014;171:1645–53. 25173451 10.1016/j.jplph.2014.08.002

[26] Zhang L, Guo X, Zhang Z et al. Cold-regulated gene *LeCOR413PM2* confers cold stress tolerance in tomato plants. Gene. 2021;764:145097. 32866589 10.1016/j.gene.2020.145097

[27] Ruggieri V, Calafiore R, Schettini C et al. Exploiting genetic and genomic resources to enhance heat-tolerance in tomatoes. Agronomy. 2019;9:22.

[28] Xin X, He SY. *Pseudomonas syringae* pv. *Tomato* DC3000: a model pathogen for probing disease susceptibility and hormone signaling in plants. Annu Rev Phytopathol. 2013;51:473–98. 23725467 10.1146/annurev-phyto-082712-102321

[29] French E, Kim B, Rivera-Zuluaga K et al. Whole root transcriptomic analysis suggests a role for auxin pathways in resistance to *Ralstonia solanacearum* in tomato. Mol Plant-Microbe Interact. 2018;31:432–44. 29153016 10.1094/MPMI-08-17-0209-R

[30] Mansfield J, Genin S, Magori S et al. Top 10 plant pathogenic bacteria in molecular plant pathology. Mol Plant Pathol. 2012;13:614–29. 22672649 10.1111/j.1364-3703.2012.00804.x PMC6638704

[31] Hu ZJ, Ma Q, Foyer CH et al. High CO_2_- and pathogen-driven expression of the carbonic anhydrase βCA3 confers basal immunity in tomato. New Phytol. 2021;229:2827–43. 33206385 10.1111/nph.17087

[32] Fragkostefanakis S, Mesihovic A, Simm S et al. HsfA2 controls the activity of developmentally and stress-regulated heat stress protection mechanisms in tomato male reproductive tissues. Plant Physiol. 2016;170:2461–77. 26917685 10.1104/pp.15.01913 PMC4825147

[33] Jia Y, Ding Y, Shi Y et al. The *cbfs* triple mutants reveal the essential functions of *CBFs* in cold acclimation and allow the definition of CBF regulons in Arabidopsis. New Phytol. 2016;212:345–53. 27353960 10.1111/nph.14088

[34] Fragkostefanakis S, Simm S, El-Shershaby A et al. The repressor and co-activator HsfB1 regulates the major heat stress transcription factors in tomato. Plant Cell Environ. 2019;42:874–90. 30187931 10.1111/pce.13434

[35] Pombo MA, Zheng Y, Fernandez-Pozo N et al. Transcriptomic analysis reveals tomato genes whose expression is induced specifically during effector-triggered immunity and identifies the Epk1 protein kinase which is required for the host response to three bacterial effector proteins. Genome Biol. 2014;15:492. 25323444 10.1186/s13059-014-0492-1 PMC4223163

[36] Zhang N, Pombo MA, Rosli HG et al. Tomato wall-associated kinase SlWak1 depends on Fls2/Fls3 to promote apoplastic immune responses to *pseudomonas syringae*. Plant Physiol. 2020;183:1869–82. 32371523 10.1104/pp.20.00144 PMC7401122

[37] Lin F, Li S, Wang K et al. A leucine-rich repeat receptor-like kinase, *OsSTLK*, modulates salt tolerance in rice. Plant Sci. 2020;296:110465. 32540023 10.1016/j.plantsci.2020.110465

[38] Jung CG, Hwang SG, Park YC et al. Molecular characterization of the cold- and heat-induced Arabidopsis *PXL1* gene and its potential role in transduction pathways under temperature fluctuations. J Plant Physiol. 2015;176:138–46. 25602612 10.1016/j.jplph.2015.01.001

[39] Kunwar S, Iriate F, Fan Q et al. Transgenic expression of *EFR* and *Bs2* genes for field management of bacterial wilt and bacterial spot of tomato. Phytopathology. 2018;108:1402–11. 29923802 10.1094/PHYTO-12-17-0424-R

[40] Ding Y, Shi Y, Yang S. Advances and challenges in uncovering cold tolerance regulatory mechanisms in plants. New Phytol. 2019;222:1690–704. 30664232 10.1111/nph.15696

[41] Li H, Ye K, Shi Y et al. BZR1 positively regulates freezing tolerance via CBF-dependent and CBF-independent pathways in Arabidopsis. Mol Plant. 2017;10:545–59. 28089951 10.1016/j.molp.2017.01.004

[42] Yoshida T, Ohama N, Nakajima J et al. Arabidopsis HsfA1 transcription factors function as the main positive regulators in heat shock-responsive gene expression. Mol Gen Genomics. 2011;286:321–32. 10.1007/s00438-011-0647-7 21931939

[43] Yeh Y, Panzeri D, Kadota Y et al. The Arabidopsis malectin-like/LRR-RLK IOS1 is critical for BAK1-dependent and BAK1-independent pattern-triggered immunity. Plant Cell. 2016;28:1701–21. 27317676 10.1105/tpc.16.00313 PMC5077175

[44] Zhang Y, Li X. Salicylic acid: biosynthesis, perception, and contributions to plant immunity. Curr Opin Plant Biol. 2019;50:29–36. 30901692 10.1016/j.pbi.2019.02.004

[45] Mou S, Gao F, Shen L et al. *CaLRR-RLK1*, a novel RD receptor-like kinase from *Capsicum annuum* and transcriptionally activated by CaHDZ27, act as positive regulator in *Ralstonia solanacearum* resistance. BMC Plant Biol. 2019;19:28. 30654746 10.1186/s12870-018-1609-6 PMC6337819

[46] Gao Y, Wu Y, Du J et al. Both light-induced SA accumulation and ETI mediators contribute to the cell death regulated by *BAK1* and *BKK1*. Front Plant Sci. 2017;8:622. 28487714 10.3389/fpls.2017.00622 PMC5403931

[47] Mosher S, Seybold H, Rodriguez P et al. The tyrosine-sulfated peptide receptors PSKR1 and PSY1R modify the immunity of Arabidopsis to biotrophic and necrotrophic pathogens in an antagonistic manner. Plant J. 2013;73:469–82. 23062058 10.1111/tpj.12050

[48] Hu C, Wei C, Ma Q et al. Ethylene response factors 15 and 16 trigger jasmonate biosynthesis in tomato during herbivore resistance. Plant Physiol. 2021;185:1182–97. 33793934 10.1093/plphys/kiaa089 PMC8133690

[49] Hu Z, Li J, Ding S et al. The protein kinase CPK28 phosphorylates ascorbate peroxidase and enhances thermotolerance in tomato. Plant Physiol. 2021;186:1302–17. 33711164 10.1093/plphys/kiab120 PMC8195530

[50] Ma Q, Liu Y, Fang H et al. An essential role of mitochondrial α-ketoglutarate dehydrogenase E2 in the basal immune response against bacterial pathogens in tomato. Front Plant Sci. 2020;11:579772. 33193523 10.3389/fpls.2020.579772 PMC7661389

[51] Ding S, Shao X, Li J et al. Nitrogen forms and metabolism affect plant defence to foliar and root pathogens in tomato. Plant Cell Environ. 2021;44:1596–610. 33547690 10.1111/pce.14019

[52] Sun M, Xu Y, Huang J et al. Global identification, classification, and expression analysis of MAPKKK genes: functional characterization of MdRaf5 reveals evolution and drought-responsive profile in apple. Sci Rep. 2017;7:13511. 29044159 10.1038/s41598-017-13627-2 PMC5647345

