# Correction: The novel leucine-rich repeat receptor-like kinase MRK1 regulates resistance to multiple stresses in tomato

**DOI:** 10.1093/hr/uhac149

**Published:** 2022-07-16

**Authors:** Qiaomei Ma, Zhangjian Hu, Zhuo Mao, Yuyang Mei, Shuxian Feng, Kai Shi

**Affiliations:** Department of Horticulture, Zhejiang University, 866 Yuhangtang Road, Hangzhou 310058, China; Department of Horticulture, Zhejiang University, 866 Yuhangtang Road, Hangzhou 310058, China; Department of Horticulture, Zhejiang University, 866 Yuhangtang Road, Hangzhou 310058, China; Department of Horticulture, Zhejiang University, 866 Yuhangtang Road, Hangzhou 310058, China; Department of Horticulture, Zhejiang University, 866 Yuhangtang Road, Hangzhou 310058, China; Department of Horticulture, Zhejiang University, 866 Yuhangtang Road, Hangzhou 310058, China

This is a **correction** to: *The novel leucine-rich repeat receptor-like kinase MRK1 regulates resistance to multiple stresses in tomato, Horticulture Research,* Volume 9, 2022, uhab088, https://doi.org/10.1093/hr/uhab088

In the originally published version of this manuscript, there was an error within Figure 3, specifically panel 3a: this should appear

**Figure 3a:**
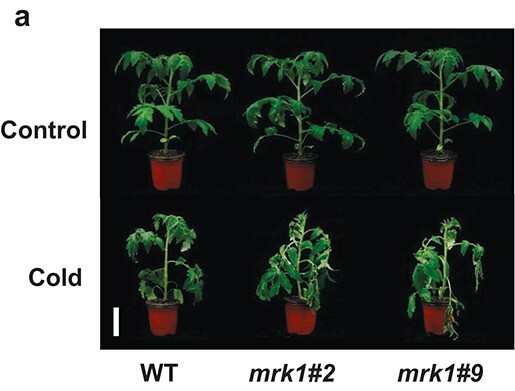


Instead of

**Figure 3a:**
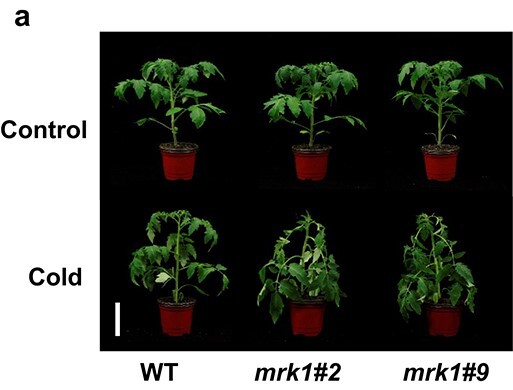


This error has been corrected (online).

