# Supplementary material for: The novel leucine-rich repeat receptor-like kinase MRK1 regulates resistance to multiple stresses in tomato
Source: Hortic Res. 2022 Jan 20;9:uhab088. doi: 10.1093/hr/uhab088 (PMC9123237; doi:10.1093/hr/uhab088)
Supplement: Web_Material_uhab088 [file web_material_uhab088.zip › Supplemental Information.docx]

**Supplementary Information**

**A novel leucine-rich repeat receptor-like kinase MRK1 regulates resistance to multiple stresses in tomato**

Qiaomei Ma, Zhangjian Hu, Zhuo Mao, Yuyang Mei, Shuxian Feng, Kai Shi^*^

**Fig. S1** Multiple resistance-associated kinase 1 (MRK1) is not involved in resistance to *Botrytis* *cinerea* (*B. cinerea*)*.*

**Fig. S2** The expression of *MRK1* and several other LRR-RLK genes in *mrk1* mutants.

**Fig. S3** Overexpression *MRK1* was unable to further enhance tomato resistance.

**Fig. S4** The expression of *MRK1* is up regulated at 1 hour post treatment with flg22.

**Fig. S5** MRK1 regulates resistance is independent of salicylic acid (SA) defense signaling.


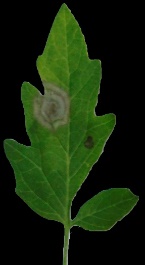

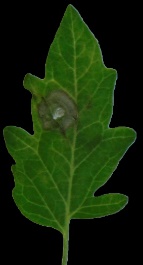


**WT *mrk1#2***

**a**

**b**

**c**

**Fig. S1: Multiple resistance-associated kinase 1 (*MRK1*) is not involved in resistance to *Botrytis* *cinerea* (*B. cinerea*)*.*** **a** The expression of *MRK1* in response to *B. cinerea* infection. **b, c** Phenotypes of WT and *mrk1* mutants leaves at 5 days post inoculation with *B. cinerea*. Bars= 1 cm. The results in **a** and **c** are presented as mean values ± SD; *n*= 5.


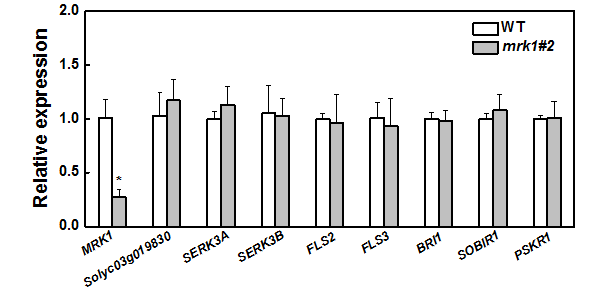


**Fig. S2: The expression of *MRK1* and several other LRR-RLK genes in *mrk1* mutants.** qRT-PCR were used to assess *MRK1* gene and several other LRR-RLK genes expression in *mrk1* mutants and WT plants. *Solyc03g01983* is the closest gene of *MRK1* in phylogenetic tree. *ACTIN2* as a normalization control. An asterisk indicates a significant difference between treatments (*P*<0.05, Tukey’s test). The results are presented as mean values ± SD; *n*= 3.

**WT OE-*MRK1#1* OE-*MRK1#3***


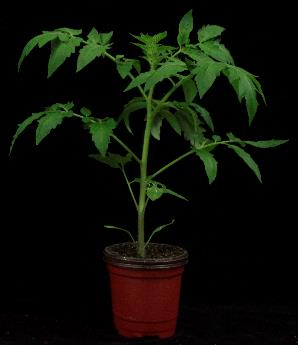

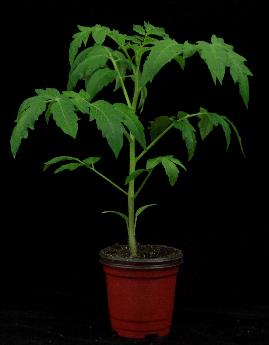

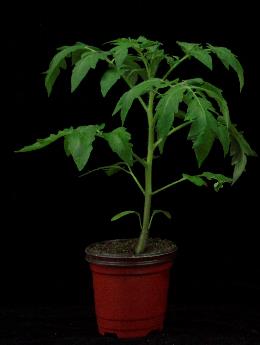

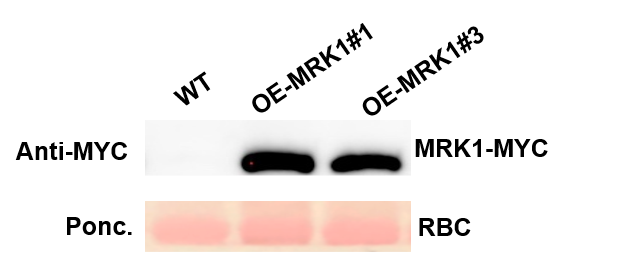

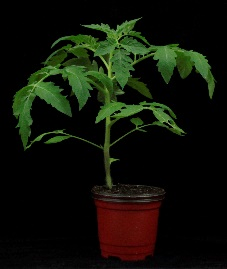

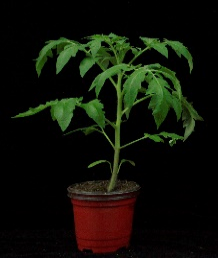

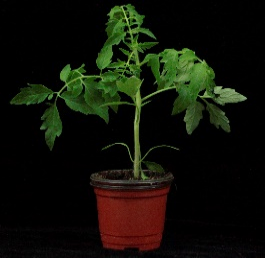

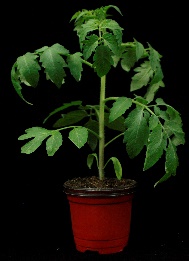

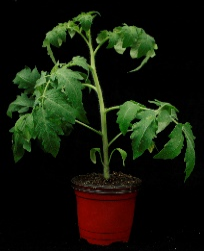

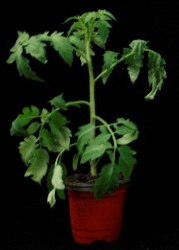


**Heat**

**Cold**

**WT OE-*MRK1#1* OE-*MRK1#3* WT OE-*MRK1#1* OE-*MRK1#3***

**WT OE-*MRK1*#1 OE-*MRK1*#3**


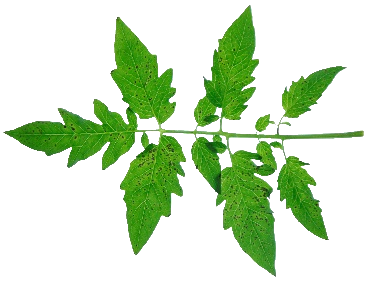

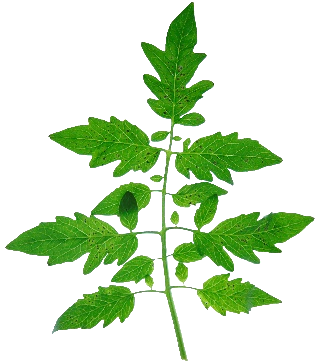

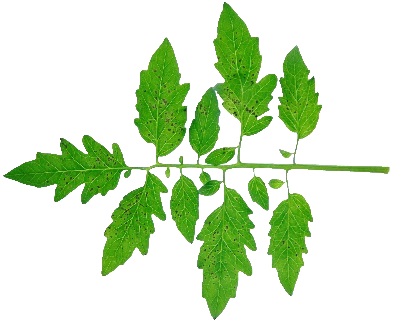


**WT OE-*MRK1*# OE-*MRK1*#3**


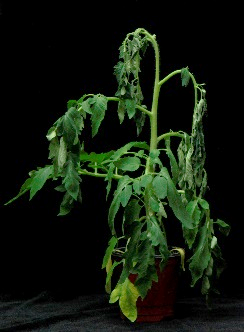

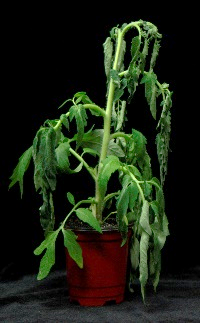

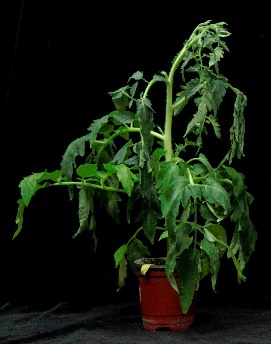

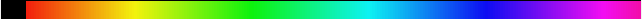


**1**

**0**


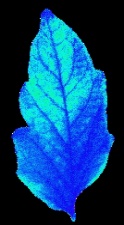

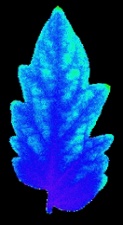

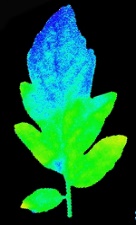

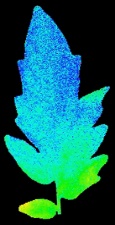

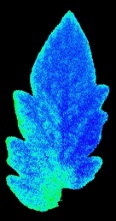

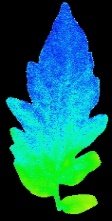


**0.51±0.04 a 0.54±0.08 a 0.55±0.06 a**

**0.61±0.05 a 0.65±0.05 0.65±0.06 a**

**a**

**c**

**b**

**d**

**e**

**f**

**g**

**h**

**Fig. S3:** **Overexpression *MRK1* was unable to further enhance tomato resistance. a** Identification of MRK1 over-expression lines by immunoblotting with an anti-MYC antibody. Rubisco (RBC) was stained with Ponceau as a protein loading control (lower panel). **b** Plants phenotype of WT plants and OE*-MRK1* plants at 4 weeks after germination. Bars= 8 cm. **c** Representative image for OE-*MRK1* and WT plants after exposure to cold temperature (Cold, 4 °C) for 7 days or high temperature (Heat, 45 °C) for 12 h. Bars= 8 cm. **d** The maximum photochemical efficiency of PSII (Fv/Fm) of OE-*MRK1* and WT plants under different temperature treatments. The color gradient scale at the right indicates the magnitude of the fluorescence signal represented by each color. Bars= 1 cm. **e** Disease symptoms of OE-*MRK1* and WT plants photographed at 5 days post inoculation (dpi) with *Pst* DC3000. Bars= 2 cm. **f** *Pst* DC3000 bacterial populations were assessed at 3 dpi. **g**. The phenotypes of tomato plants at 10 dpi with *R. solanacearum*, and *R. solanacearum* bacterial population (**h)** in plants were assessed at 10 dpi. Bars= 8 cm. The results in **d, f** and **h** are presented as mean values ± SD; *n*= 5. An asterisk indicates a significant difference between treatments (*P*<0.05, Tukey’s test). These experiments were performed twice with similar results.

**Fig. S4: The expression of *MRK1* is up regulated at 1 h post treatment with flg22.** An asterisk indicates a significant difference between treatments (*P*<0.05, Tukey’s test). The result is presented as mean values ± SD; *n*= 3.

**Fig. S5: MRK1 is independent of salicylic acid (SA) defense signaling. a** The *Pseudomonas syringae* pv. *tomato* (*Pst*) DC3000-induced SA accumulation is not affected by *mrk1* mutation. WT and *mrk1* mutants were inoculated with *Pst* DC3000 or mock control, and leaf samples were collected 24 h post-inoculation (hpi) with *Pst* DC3000. **b** Expression of SA biosynthesis marker gene *PAL4* was assessed at 12 hpi. **c, d** Expression of SA defense signaling marker genes (*PR1* and *PR4*) was assessed at 12 hpi. An asterisk indicates a significant difference between treatments (*P*<0.05, Tukey’s test). The results are presented as mean values ± SD; *n*= 3.
