# Supplementary material for: The novel leucine-rich repeat receptor-like kinase MRK1 regulates resistance to multiple stresses in tomato
Source: Hortic Res. 2022 Jan 20;9:uhab088. doi: 10.1093/hr/uhab088 (PMC9123237; doi:10.1093/hr/uhab088)
Supplement: Web_Material_uhab088 [file web_material_uhab088.zip › Table S2.pdf]

Table S2. The primers used in this study

Gene	Accession No.	Primer pairs	Assay
MRK1	Solyc01g105080	F: 5'- TCCCATCGCTACTCAACGAA -3'<br>R: 5'- CGGTGGAGCAGAATGTGAAG -3'	qPCR
		F: 5'- GATTGAAGTTGACTGATTAAAACCG -3'<br>R: 5'- AAACCGGTTTTAATCAGTCAACTTC -3'	pCAMBIA1301
		F: 5'- CTTACTGGGAAACTCAAGGC -3'<br>R: 5'- TGGATAATTTTGAACTATTT -3'	mutation detection
		F: 5'- CCCttaattaacATGGGTTCATCTAGTCTCTT -3'<br>R: 5'- AAAggcgcgcccTCGAGAGGGAGTTGCAGTTGC -3'	BiFC-nYFP
		F: 5'- ggggacaagtttgtacaaaaaagcaggctttATGGGTTCATCTAGTCTCTT -3'<br>R: 5'- ggggaccactttgtacaagaaagctgggtcTCGAGAGGGAG TTG CAGTTGC -3'	pDONR-Zeo
-	Solyc03g019830	F: 5'- TGCTTTGTTGCAGCTGAGAG -3'<br>R: 5'- CATGGCCATCAGAACACGAA -3'	qPCR
FLS2	Solyc02g070890	F: 5'- ATCCCAGAGAGCATGACGAG -3'<br>R: 5'- GCAACAAGTTGCTGGAAACG -3'	qPCR
		F: 5'- CCCttaattaacATGATGATGTTAAAGACAGTTG -3'<br>R: 5'- AAAggcgcgcccATCTTTTACCAAATGAGAAG -3'	BiFC-cYFP
		F: 5'- ggggacaagtttgtacaaaaaagcaggctttATGATGATGTTAAAGACAGTTG -3'<br>R: 5'- ggggaccactttgtacaagaaagctgggtcATCTTTTACCAAATGAGAAG -3'	pDONR-Zeo
FLS3	Solyc04g009640	F: 5'- AAATCCCTGATGGCATTGGC -3'<br>R: 5'- ACCGTTCCACATTTCACCAC -3'	qPCR
SERK3A	Solyc10g047140	F: 5'- CCCttaattaacATGGATCAGTCGGTGTTGGC -3'<br>R: 5'- AAAggcgcgcccTCTTGGCCCTGACAACTCATC -3'	BiFC-cYFP

		F: 5'- ggggacaagtttgtacaaaaaagcaggctttATGGATCAGTCGGTGTTGGC -3'<br>R: 5'- ggggaccactttgtacaagaaagctgggtcTCATCTTGGCCCTGACAACTC -3'	pDONR-Zeo
SERK3B	Solyc01g104970	F: 5'- CCCttaattaacATGATGGATCAATGGGTCTT -3'<br>R: 5'- AAAggcgcgcccTCTTGGCCCTGATAACTCATC -3'	BiFC-cYFP
		F: 5'- ggggacaagtttgtacaaaaaagcaggctttATGATGGATCAATGGGTCTT -3'<br>R: 5'- ggggaccactttgtacaagaaagctgggtcTCTTGGCCCTGATAACTCATC -3'	pDONR-Zeo
BRI1	Solyc04g051510	F: 5'- TACTGACCGGGAAGCGATAC -3'<br>R: 5'- AATCCCTCCAAACTCCAGCA -3'	qPCR
SOBIR1	Solyc06g071810	F: 5'- ATCTGCTGTGCTCTGTTCCT -3'<br>R: 5'- ATTTCAAGCGATGCCACTCC -3'	qPCR
PSKR1	Solyc01g008140	F: 5'- CCGTGGGTTAGTTCCTGATT -3'<br>R: 5'- GAAAGGGACGAAAGGCTATG -3'	qPCR
CBF1	Solyc03g026280	F: 5'- GTGACTTCGTGGATGAGGAG -3'<br>R: 5'- AGGCATCAGTTTCCACACAA -3'	qPCR
HsfA1a	Solyc08g005170	F: 5'- TAGCTGAAGGCAGCAAGAAA -3'<br>R: 5'- CTGCCTCATTTATCCCAGGT -3'	qPCR
OLP	Solyc11g044390	F: 5'- TCCTAATGACTTGTCCGGATTT -3'<br>R: 5'- AGTATCACTAGGGCAAGCAAATA -3'	qPCR
LPSE	Solyc04g077180	F: 5'- CAGCATTCTGTGGGCTATAC -3'<br>R: 5'- CCGAAGAAGAAGAGGTTTCC -3'	qPCR
PAL4	Solyc09g007890	F: 5’- AATCGCGATGGCTTCTTACT -3’<br>R: 5’- CCCAACGAATTCACATCTTG -3’	qPCR
PR1	Solyc01g106620	F: 5’- CTCGGTACGTCTTGGTTGTG -3’<br>R: 5’- TCCAGTTACCTGGTGGATCA -3’	qPCR
PR4	Solyc00g174340	F: 5’- TAGTCTGGCGCAACTCAGTC -3’<br>R: 5’- TGCAAGAAATGAACCACCAT -3’	qPCR
ACTIN2	Solyc03g078400	F: 5'- TGGTCGGAATGGGACAGAAG -3'<br>R: 5'- CTCAGTCAGGAGAACAGGGT -3'	qPCR
B. cinerea ACTIN	-	F: 5'- GGTAACATTGTTATGTCTGG -3'<br>R: 5'- CTTGACCTTCATCGACG -3'	qPCR
